# Staying active through life’s shifting seasons: a qualitative study of community-dwelling older adults’ experiences of habit formation and physical activity in later life

**DOI:** 10.1186/s11556-025-00393-8

**Published:** 2025-11-27

**Authors:** Åsa Karlsson, Sara Lundell, Marit Solbjør, Beatrice Pettersson

**Affiliations:** 1 Department of Community Medicine and Rehabilitation, Umeå University, Umeå, Sweden; 2 Department of Public Health and Nursing, Faculty of Medicine and Health Science, Norwegian University of Science and Technology, Trondheim, Norway

**Keywords:** Aged, Physical activity, Exercise, Habits, Behaviour change

## Abstract

**Background:**

Regular physical activity is essential for healthy aging, yet sustaining long-term engagement remains a challenge for many older adults. Emerging research highlights habit formation as a promising mechanism for maintaining physical activity, particularly when supported by contextual cues and digital technology.

**Objective:**

This qualitative study explores how community-dwelling older adults incorporate physical activity into their daily lives, focusing on the processes of habit formation and maintenance, and the role of technology in supporting these behaviours.

**Methods:**

Data were collected through three focus group interviews, one dyadic interview, and two workshop sessions conducted in Umeå, Sweden. Fifteen participants (mean age 73.3 years) with previous experience with regular physical activity contributed to the study. Reflexive thematic analysis was used to interpret the data, following Braun and Clarke’s six-phase approach.

**Results:**

The analysis generated one overarching theme “Staying active through life’s shifting seasons” which captures how participants adapted their physical activity routines in response to aging, health changes, and life transitions. Two interwoven subthemes were identified: “Building the habit scaffold” and “Cultivating a movement mindset”. The first describes how participants constructed supportive frameworks for activity through routines, environmental adjustments, social contracts, and digital technology. The second highlights internal strategies such as emotional reframing, identity alignment, and the development of a personal philosophy around physical activity. Participants emphasized the importance of flexibility, self-awareness, and emotional engagement in sustaining activity. Technology, including smartwatches and fitness apps, was described as both a motivational aid and a feedback system, reinforcing routines and providing a sense of accountability. Seasonal variation and life events were common disruptions, requiring participants to renegotiate their habits and find new forms of physical activity.

**Conclusions:**

Sustaining physical activity in later life is a multifaceted and adaptive process, maintained through the interplay of external scaffolding such as routines, cues, and supportive structures and internal orientations rooted in identity, emotion and meaning. Interventions that are practical, motivational, and flexible are likely to be most supportive. Digital technology can offer valuable support if embedded into personally meaningful and adaptable routines.

**Trial registration:**

NA

**Supplementary Information:**

The online version contains supplementary material available at 10.1186/s11556-025-00393-8.

## Background

Engagement in regular physical activity and structured exercise is a cornerstone for promoting healthy aging [1]. Physical activity can help mitigate age-related physiological and cognitive decline; reduce the risk of chronic diseases such as cardiovascular disease, type 2 diabetes, and dementia; and enhance overall quality of life and functional independence among older adults [2]. Recent international consensus emphasizes that tailored, multicomponent exercise programs incorporating aerobic, resistance, balance, and flexibility training are essential not only for disease prevention but also for maintaining mobility, independence, and resilience among older adults [2]. Physical inactivity and sedentary behaviour are now recognized as key modifiable risk factors linked to frailty, disability, and premature mortality [3]. This highlights the urgent need for effective strategies to support older adults in initiating and maintaining regular physical activity and exercise routines throughout later life. Despite these well-documented benefits, a large proportion of older adults remain insufficiently active [4, 5].

To effectively promote and sustain physical activity among older adults, it is essential to understand the psychological and contextual factors that influence behaviour over time. While many physical activity and exercise interventions successfully initiate behaviour change, maintaining regular behaviour remains a challenge [6]. Qualitative studies have provided important insights into how older adults perceive and experience engaging in physical activity. In a thematic synthesis, Franco et al. [7] identified social influences, health beliefs, physical limitations, and self-efficacy as key factors shaping participation. In another qualitative synthesis, Meredith et al. [8] used the COM-B framework to map factors influencing participation among adults aged 70 and older. The synthesis aligns with results from Franco et al. [7] and further demonstrates that engagement reflects the interplay of capability, opportunity, and motivation. While this literature illustrates the multifaceted nature of participation, less is known about how older adults develop and sustain physical activity habits over time.

Addressing this challenge has prompted growing interest in innovative approaches, including the use of digital technologies, to support behavior change and long-term adherence. Evidence suggests that digital physical activity interventions are effective at increasing physical activity in older adults [9] Although further research is needed in adults aged 65 years and over exclusively, and with longer-term follow-up [9]. It also remains unclear whether technologies can reliably increase motivation for physical activity in this population, which motivational dynamics are primarily activated by such tools, and which types of technology are most effective in enhancing motivation [10].

Complementary to technological solutions, increasing attention has been given to the role of habit as a key automatic process in sustaining health-related behaviours. Unlike self-regulatory strategies, which rely on conscious effort and limited psychological resources, habits can support maintenance by reducing the cognitive burden of repeated action [11, 12]. Habit formation is positively associated with physical activity engagement and has become a central concept in recent health behaviour research [13, 14]. Habits are formed through repeated execution of a behaviour in response to consistent contextual cues. Through this repetition, a cognitive association between the cue and the behaviour is established, which can later trigger the behaviour automatically when the cue is encountered [15]. This process, often referred to as cue-behaviour repetition, is foundational to habit development. Most empirical studies have focused on how repeated pairing of cues and behaviours leads to habit formation, with consistent evidence supporting this direction of influence [11, 16]. There is a growing number of interventions aimed at promoting sustained physical activity through habit-based strategies, and evidence for their effectiveness is accumulating. Systematic reviews and meta-analyses have shown that habit strength is positively associated with physical activity engagement and that habit formation interventions can produce small to moderate improvements in behaviour [13, 17]. However, the vast majority of this research has been conducted in younger or mixed-age populations. In particular, little is known about how older adults themselves experience the process of creating and sustaining new habits. This gap is notable given the particular challenges and opportunities of later life, where ageing, health changes, and shifting routines may shape habit processes differently. To address this, the present study explores how community-dwelling older adults incorporate physical activity and exercise into their daily routines, focusing on the formation and maintenance of habits and the role of technology as a potential support for these practices.

## Methods

A qualitative study design was used, consisting of focus groups (FG), a dyadic interview (DI), and one workshop (W). The Standards for Reporting Qualitative Research (SRQR) was used as a guideline to ensure transparency in reporting [18].

### Study context

The study was conducted in Umeå, northern Sweden (latitude 63.8° north), where snow or ice typically covers the ground during the main part of winter (December to April). Umeå is a Scandinavian university city with approximately 130,000 inhabitants and a strong emphasis on sustainable urban planning and public health promotion. The municipality promotes active transport and prioritizes cycling infrastructure, including snow clearance of bike lanes during winter. As a result, cycling remains a common mode of transport throughout the year. In addition, cross-country skiing is a widely practiced form of physical activity during the winter months, supported by accessible trails and local culture.

### Participants

Participants were recruited through two main channels. Members of a major local senior organization received an email invitation and were encouraged to contact the principal investigator if interested. Additionally, participants from physical activity groups were provided with both oral and written information in connection with one of their regular training or walking activities and were similarly invited to reach out to the principal investigator to express interest. Individuals were eligible to participate if they were: 65 years of age or older, living independently in their own homes, and able to read, write, and speak Swedish. The inclusion criterion of 65 years or older was chosen as this age is recognized in Swedish health care and municipal elder care services as the point at which individuals are categorized as older adults in gerontological and geriatric contexts. Seventeen participants reported interest in participating in the study. Two persons dropped out before data collection; one was not available the suggested dates, and one accepted but did not show up.

All included participants (*n* = 15) were born in Sweden, with a mean age of 73 years, and had experience of regular physical activity in adulthood, mainly walking, bicycling, group fitness classes, gyms, and cross-country skiing (Table 1).

**Table 1:** Participant characteristics

Variable	Participants (*n* = 15)
Age (years), mean ± SD (min-max)	73.3 ± 6.1 (65–86)
Women, n (%)	9 (60.0)
Use of internet or applications on smart technology, n (%)	
≤4 days per week	0
5 days per week	1 (6.7)
6 days per week	0
Every day	14 (93.3)
Self-rated physical activity, n (%)	
Mild/moderate physical activity	
<30 min	0
30–60 min	1 (6.7)
60–90 min	5 (33.3)
90–150 min	3 (20.0)
>150 min	6 (40.0)
Strenuous physical activity	
<30 min	2 (13.3)
30–60 min	4 (26.7)
60–90 min	1 (6.7)
90–120 min	4 (26.7)
>120 min	4 (26.7)
Sedentary behaviour, n (%)	
≤3 h	0
4–6 h	12 (80.0)
7–9 h	2 (13.3)
10–12 h	1 (6.7)
≥13 h	0

### Data collection

Data for the current study comprise three focus groups and one dyadic interview, all conducted between February and December 2024. The focus groups each involved three to five participants. All interviews were held at Umeå University, with two conducted in UmeHealth Lab, a home-like environment designed to create a relaxed and familiar atmosphere for discussion. Participants were offered multiple date options, and the date with the highest availability was selected. To facilitate participation, parking permits were arranged for those traveling by car, and in one case, taxi transportation was provided due to mobility limitations. Two of the focus groups were carried out within a workshop series aimed at developing digital support for habit formation to promote physical activity among older adults. Participants from the first and second focus group also took part in an additional workshop session with an in-depth discussion on cues for exercise and how these cues could be supported digitally. The transcript from that session was also included in the analysis to enrich the data. Additionally, owing to logistical constraints, one dyadic interview was also conducted. All interviews were audio recorded, transcribed verbatim, and lasted between 74 and 107 minutes. The participants’ background characteristics were collected via a short questionnaire.

The focus groups and workshop were moderated by BP, with extensive experience in facilitating group interviews. ÅK and SL attended as co-moderators and documented group dynamics and nonverbal cues. The dyadic interview was conducted by BP alone. Each session began with a brief introduction to the study and a reminder of the purpose of participation. To ensure a shared understanding, participants were also presented with definitions of physical activity and exercise, recommendations for physical activity for adults aged 65 and over, and distinctions between behaviour change and habit formation before the discussion started. At the start of each session, participants introduced themselves and shared a personal interest to create a relaxed atmosphere, and the moderator clarified that both successful and less successful experiences were valuable for the discussion. A semi-structured interview guide was used to ensure consistency across interviews while allowing flexibility for participants to elaborate on issues of personal relevance (Appendix 1). The guide included topics such as participants’ previous experiences of trying to establish routines for physical activity and exercise (e.g., “Could you tell us about your previous experiences of trying to establish a regular routine for exercise or physical activity?”), factors that support or hinder the development of sustainable habits (e.g., “In your experience, what is important for building and maintaining a sustainable exercise habit?”), and situational or emotional cues that may trigger physical activity (e.g. “Can you describe any situations or circumstances that usually help, or might help, you to get started with physical activity or exercise?”). It also explored the potential role of digital technologies in supporting behaviour change, including preferences regarding reminders, goal tracking, and desired functionalities in digital technology. In line with established focus group methodology [19], the moderators fostered open, reflective dialogue, encouraged participants to share and build upon each other’s experiences, and ensured that all voices were heard by actively redistributing speaking time when imbalances occurred and using prompting questions to invite quieter participants and elicit diverse perspectives.

### Analysis

Data were analysed via reflexive thematic analysis, following the six-phase process described by Braun and Clarke [20]. The analysis was facilitated by the software MAXQDA 2022. The analysis was conducted by a team of four researchers (ÅK, SL, MS, BP), all with substantial experience in qualitative research. First, ÅK, BP, and SL independently coded a portion of one interview to assess coding consistency and enhance analytic rigour. Initial inductive coding was led by BP, generating both semantic and latent codes. These were reviewed and refined collaboratively in multiple rounds with all the authors. The first author wrote memos to document analytical insights and evolving ideas. Theme development was iterative and dialogical rather than linear. Initially, three themes were handled as parallel and with equal analytical weight. However, through ongoing team discussions and critical reflection, we identified participants’ engagement with physical activity not as static but as a process evolving over time. This led to a conceptual reorganisation: the three themes were reconsidered as one overarching theme describing the dynamic and lived nature of habit formation, with the other two reframed as sub-themes. The final thematic structure captured a broad narrative pattern in the main theme and more specific but interconnected strategies for integrating physical activity into everyday life in the sub-themes.

### Researcher reflexivity

The author team comprised three physiotherapists (ÅK, BP, and SL) (two specialising in older adults’ health (ÅK and BP), one with expertise in behaviour change related to physical activity (BP ), and one health sociologist (MS). All authors had prior experience with qualitative research; three had long-standing experience (SL, MS, and BP), and one (MS) had substantial expertise in reflexive thematic analysis. The sociologist (MS) brought complementary expertise in health services research and health sociology, strengthening attention to contextual, structural, and interactional dynamics in care.

To support reflexivity, all authors engaged in regular analytic discussions and remained mindful throughout the process of how our professional backgrounds, perspectives on physical activity among older adults, and prior familiarity with the field might influence our interpretations. The thorough inductive coding process also helped bracket and move beyond preconceptions. Divergent readings, based on insider and outsider perspectives, were seen as opportunities to explore alternative understandings of the data, reaching analytical consensus through discussion among all authors.

## Results

The analysis generated an overarching main theme ‘Staying active through life’s shifting seasons’, which captures how older adults navigated and adapted their physical activity routines across the changing contexts of aging, health, and everyday life. Rather than representing static behaviour, physical activity emerged as a dynamic and evolving process shaped by transition, disruption, and continuity.

Within this overarching pattern, two interwoven sub-themes were identified, ‘Building the habit scaffold’ and ‘Cultivating a movement mindset’, each illuminating different yet complementary ways in which participants sustained their activity over time (Appendix 2). One focused on the practical frameworks that supported habit formation, and the other focused on internal perspectives and emotional strategies. Together, often overlapping, they show how older adults sustained physical activity over time by combining planning and structure with motivation, flexibility, and personal meaning.

### Staying active through life’s shifting seasons

This theme represents a narrative arc through participants’ experiences, showing how physical activity was adapted over the life course in response to ageing, changing health, and shifting roles. Rather than fixed routines, activity was shaped by ongoing adjustments to new circumstances.

Participants expressed a deep awareness of how ageing altered the very terms of physical activity, describing it as a continuous process of both *knowing and not knowing the body* and of constantly relearning what it could do. Episodes of vulnerability, such as illness, injury, or falls, often deepened this awareness, prompting closer listening to bodily signals, limits, and needs. Rebuilding confidence was not only about physical recovery but also about restoring trust in the body’s ability, with physical activity itself described as a way to reinforce capability even when the body felt unpredictable or fragile. For some, living within current capacities meant letting go of past identities such as “runner” or “tennis player” and finding meaning in new forms of activity. *Adapting to setbacks and changing conditions* was therefore a common experience, as participants frequently had to adjust their routines in response to health events, environmental barriers, or logistical changes. Injuries, surgeries, chronic illness, or acute conditions could interrupt activity for long periods, leading to a search for alternatives that matched current abilities, for example replacing high-impact exercise with gentler options or using walking aids to stay mobile outdoors.

*“These days*,* I do more low-intensity exercise than I used to. For a while I thought I was just out of shape*,* that I just needed to push harder…It’s about finding the things you can do. You realize that you’re mortal. It’s part of the deal.” (Man 1*,* FG1*,* 81 years)*.

The participants also described how seasonal changes could disrupt continuity. Winter was seen as an opportunity for activity, with cross-country skiing being particularly appreciated as both low- and high-intensity activity. Ice covered surfaces or cold weather could limit opportunities for activity, especially when combined with or concern from family members of them bicycling or fear of falling.

*“The fear has increased because the winters have gotten worse. It’s so damn slippery all the time…It feels like it’s only a matter of time before you fall and break something.” (Woman 1*,* FG3*,* 70 years)*.

Everyday life events could also challenge routines, and several participants noted how difficult it was to return to regular activity after a break, even when it was short. Moving or losing access to familiar infrastructure often also meant starting over in a new context. Despite these shifts, many worked on being physically active within their new circumstances, sometimes reframing goals to focus on what was possible rather than what was lost. One participant offered a critical reminder that routines should serve life, not the other way around:

*“Everything has its time in life…I mean*,* things happen in life that make it hard to stick to routines. You sound like a role model in regard to routines [addresses a man in the group]. But really*,* it’s about not thinking*,* “Oh no*,* now I have to manage that too.” We’re not going to die just because we can’t keep it up. Life is what matters.” (Woman 2*,* FG1*,* 73 years)*.

Major life transitions added yet another layer of disruption and *reframing of physical activity.* Participants described how transitional phases such as retirement, changes in caregiving roles, or children leaving home reshaped their opportunities and motivation for physical activity. Retirement could open up for more time for physical activity which could be experienced as liberating, with renewed engagement in physical activity and exercise. However, retirement could also remove a familiar structure and rhythm of the everyday life, sometimes leading to a loss of momentum for physical activity. Changes in social networks constituted another transitional phase that also challenged continuity. Friends moving away or group activities ending could disrupt long-standing routines. Some participants replaced these with new activities after a period of adjustment, whereas others withdrew fully from previous habits.

*“We were about ten people who played badminton and went to the sauna together*,* but gradually the friends I played with and enjoyed spending time with stopped coming. So*,* I think everything has had its time.” (Man 1*,* DI*,* 80 years).*

### Building the habit scaffold

This sub-theme captures how the participants worked to establish and maintain regular physical activity through practical strategies, resources, and external support. Rather than relying solely on motivation, they actively constructed a scaffold of routines, tools, and adjustments that made physical activity both possible and sustainable.

Participants emphasized that forming a habit required *having the right conditions* in a physical, social, and logistical sense. Professional support, such as physiotherapy or structured programmes, was particularly valued during rehabilitation or when new activities were started, although transition to independent exercise could be challenging. Access to facilities was another important condition. Long distances or limited local options made consistency harder, whereas living close to a gym or having equipment at home reduced barriers.

*“A gym has opened nearby*,* which makes it easier to go. — it’s just across the courtyard”. (Man 1*,* DI*,* 80 years).*

This was especially highlighted by participants who lived in rural areas, who also noted how digital solutions, to some extent, could compensate for limited availability of options.

Another prominent strategy was *finding what worked through trial and error*. The participants experimented with different activities, environments, and routines to match their preferences, bodies, and daily lives. Gradually increasing intensity and duration was seen as key to building confidence and avoiding bodily and motivational setbacks. In this process, social norms for age-appropriate activity and gender roles contributed to shaping what felt accessible. For instance, some preferred inclusive environments such as senior fitness classes while others avoided overly competitive settings or initiatives “*for old people*”.

*“There’s a kind of threshold with things like this. I go to water aerobics at the community centre*,* and when I invite friends they say*,* ‘Isn’t that just for old people?’ I tell them*,* ‘Well*,* you’re old too.’ But there’s this stigma — like going there means you’re really old*,* so you just can’t go.” (Man 2*,* DI*,* 72 years)*.

A key aspect of the trial-and-error process was the need to find joy, meaning, and low-threshold opportunities. Valuing the freedom to explore physical activity indoors and outdoors, as well as combining structured and informal activities, allowed variance rather than committing to one type of activity. The participants expressed that the process of “*finding your element”* was not always straightforward. Experimentation was essential to identify sustainable ways to stay active, especially when previous routines no longer worked.

*“The local gym offers everything from intense training to yoga and swimming. Having all those options in one place makes it easier.” (Woman 1*,* FG1*,* 71 years)*.

Creating *a predictable structure* was key to maintaining regular physical activity. Participants emphazised the value of having set days, times, and rhythms that signalled when it was time to move, which helped reduce the cognitive load of decision-making and supported consistency even when motivation dipped. For many, activity became part of a weekly schedule, with certain days reserved for exercise and others left open for recovery or spontaneity. At the same time, the participants stressed the importance of being able to adapt, e.g., shifting a session in response to family responsibilities, fluctuating energy levels, or weather. Consistency was further supported by anchoring activity to specific times of day or by external triggers, such as reminders from a smartwatch, encouragement from a partner, or a dog waiting for its walk, which all helped integrate physical activity into the flow of everyday life.

*Social contracts* also supported habit scaffolding. Accountability to friends, family members, and/or group exercise instructors helped uphold routines. Some trained with a partner or committed to a specific gym schedule with someone else, which created a sense of obligation. Others described how regular participation in group activities fostered a sense of belonging and mutual encouragement. Digital technology such as training apps extended this support, allowing users to connect, share progress, and engage in friendly competition.

*"We meet up through the cycling app*,* chat at checkpoints*,* and arrange monthly gatherings." (Man 3*,* FG1*,* 73 years)*

These technologies functioned as *feedback systems* rather than passive trackers, becoming interactive companions that provided prompts, feedback, and motivation.

For some, the app acted as a daily or weekly reminder to move when their step count was low, while gamified features such as streaks and badges made activity more engaging and, at times, playful. Positive feedback such as progress bars or celebratory sounds sparked pride and satisfaction, and many participants described joy in seeing their efforts accumulate over time. At the same time, the relationship was not always straightforward, as negative prompts or “snarky” messages could lead to guilt or frustration. Yet the objectivity of the data also provided a sense of self-accountability, with one participant noting, “*you can’t escape the data*”, and supported reflection and adjustment in line with physical capacity and long-term goals.

*“Man 1 (67 years): You might think: Wow*,* I’ve walked 8*,*000 steps*,* that’s great.*

*Woman 1 (82 years): Now I can relax a bit tonight.*

*Man 1 (67 years): Right*,* that was good*,* with the calories and all that… And the heart on the app glows even redder.*

*Woman 1 82 years): And suddenly it just feels better. [laughs]” (FG2)*

Technology could also serve as a tool for safety and reassurance. Participants mentioned using wearable devices with fall detection features that could alert family members or emergency care. Having the mobile phone with them or GPS sharing with children or partners helped some feel more secure when exercising alone, especially outdoors or in challenging terrain.

*“When I go cycling*,* I always message the kids. That way they can track me – just in case.” (Man 1*,* FG 2*,* 67 years)*.

Thus, rather than giving up on outdoor activities, participants used technology to maintain autonomy and continuity in their routines.

### Cultivating a movement mindset

This sub-theme captures the importance of internal processes, ways of thinking and feeling that sustained engagement among the participants. Everyday opportunities for movement were redefined as meaningful actions. Rather than relying solely on discipline or cues, participants shaped their thinking, attitudes, and emotions around activity to strengthen commitment and foster meaning, enjoyment, and purpose.

Engaging in physical activity was both a rational and emotional choice, rooted in knowledge of bodily needs and a desire to have a good quality of life. Making sense of the value of physical activity often involved sorting through health information, societal expectations, and scientific claims, leading to a more personal, sustainable understanding. Many framed physical activities as a long-term *investment in one’s health* and independence, linked to tangible goals such as avoiding surgery, managing weight or pain, and staying active with grandchildren.

*“I want to be able to crawl on the floor and play and to support my family. It’s also about saying yes to life. And for that*,* you need strength.” (Woman 2*,* FG1*,* 73 years)*.

This investment orientation extended to different forms of physical activity, and many had built a personal repertoire of sustainable and beneficial activities. In addition to being an investment in health, physical activity was framed as a form of personal agency and as taking control of one’s health. Especially for those who had experienced loss of e.g., mobility, health, or a loved one, physical activity could become a way to re-establish a direction in life and foremost a way to reassert one’s ability to influence life circumstances. This sense of ownership was tied to small but deliberate actions: choosing to walk rather than drive, using the stairs carrying groceries, or deciding to keep mowing the lawn yourself. It could also be using small tricks or prompts to encourage activity, like placing the remote control to the television across the room. For some, this mindset emerged as a result of previously developed habits. For others, it was a deliberate strategy to make activity natural and low effort.

*“If it snows all day*,* I go out [shovelling] two or three times rather than waiting. I also always park far away when I go shopping. That’s become part of my mindset*,* to build movement into everyday life.” (Man 2*,* FG 1*,* 70 years)*.

For a number of participants, the role of movement had deepened to the point of *being a basic need*, akin to eating or sleeping with bodily cues such as restlessness, discomfort, or a lack of energy signalling the need to move.

*“It’s simply that you’re so used to going outside that if there’s a whole day when you don’t even poke your nose out*,* you just don’t feel well. It’s so fundamental somehow that you have to get out for a bit.” (Man 2*,* DI*,* 72 years).*

Maintaining an active lifestyle was also tied to a sense of *responsibility*,* self-regulation*,* or emotional commitment*, either to oneself or others. Participants spoke of internal expectations and a desire to live up to personal standards or an active identity. This commitment was often reinforced through internal dialogues like talking oneself into getting up to move or imagining the disappointment of skipping a session. This inner negotiation, described as *“having both a devil and an angel on the shoulder”*, reflected an ongoing process of self-regulation: balancing goals, values, and the realities of ageing to preserve consistency and autonomy.

Negative *emotional responses* such as guilt, shame, or frustration sometimes influenced behaviour particularly when participants felt they had not lived up to their expectations. Some avoided healthcare professionals out of embarrassment when they had not followed recommendations of physical activity or rehabilitation programmes. Moreover, positive feelings such as pride and a sense of accomplishment were important motivators, especially when activities felt mundane or demanding. Descriptions of calm, mental clarity, or physical satisfaction after completing physical activity were common, and these sensations helped reinforce motivation. Activities that brought joy, such as dancing, being outdoors, or walking in nature were especially valued. Add-ons like music, audiobooks, or gamified apps could also make physical activity or exercise more appealing.

*“It really lifts your spirits when you’re outside with the forest and the birds. It’s just wonderful…and you can listen to things [e.g.*,* audiobooks]” (Woman 2*,* FG 3*,* 69 years)*.

These emotional and experiential aspects were not mutually exclusive but often coexisted, together influencing motivation and the ongoing process of habit formation in physical activity.

## Discussion

This study explored how community-dwelling older adults sustain physical activity over time, showing it as an evolving practice shaped by bodily changes, life transitions, and emotional dynamics. The overarching theme, ‘Staying active through life’s shifting seasons’, captures how habits were repeatedly renegotiated rather than linearly formed. Our findings suggest that physical activity in later life involved phases of disruption and reorientation. This interpretation resonates with theoretical accounts such as the habit discontinuity hypothesis, which highlights how major life changes can destabilise routines and create openings for new behaviours [21].

Retirement provided a vivid example of such discontinuity, described by participants as both an opportunity and a disruption in relation to physical activity. This duality echoes previous findings that reported changes in physical, social, and intellectual activity following retirement, although the direction and stability of these changes differed across individuals [22]. Similarly, Barnett et al. [23] noted that while leisure-time physical activity often increases post-retirement, overall activity levels may vary. The ability of the participants in our study to sustain activity during such transitions was often contingent on whether they could draw upon meaningful routines or personal motivations. This underlines the importance of being aware of the impact of major life transitions on habits for physical activity and exercise.

Participants emphasized physical activity as a way to maintain identity and continuity amid bodily changes and shifting life roles, though some felt that certain forms of exercise not ‘were for them’, reflecting how age and gender shaped what felt accessible. Similar findings were reported by Bennett et al. [24], who reported that older women used physical activity to affirm independence while also distancing themselves from images of frailty. González-Calvo et al. [25] likewise found that older men used training to adjust their sense of self in relation to bodily changes. Beyond physical function, physical activity can support autonomy, quality of life [26], and help preserve a sense of self despite age-related cognitive changes [27]. Together, these studies show that physical activity can be an arena where older adults reinterpret who they are and how they age.

The participants frequently adapted their physical activity routines in response to seasonal changes. While some embraced winter activities, others reduced movement due to icy ground, cold weather, or fear of falling. These experiences echo Canadian and Nordic research showing that seasonal barriers can be a risk for inactivity [28, 29] contrasting with southern Europe where such variation is minimal [30], highlighting the greater demands in fluctuating climates. Given that physical activity levels already tend to decline with age [31], our findings suggest that seasonal barriers can further challenge the continuity of physical activity habits among older adults.

The practical strategies used to support maintenance of physical activity and exercise illustrate what we conceptualized as ‘Building the habit scaffold’. Maintaining activity often involved embedding it in daily life through predictable structures, fixed times, or prompts such as digital reminders. This resonates with habit theory, which emphasizes repetition in stable contexts as a route to automaticity [11, 12]. While few participants described their behaviour as fully automatic, many reported habitual instigation, where cues or bodily sensations triggered action. Evidence supports this process, where nonconscious mechanisms contribute to activity beyond conscious intentions [12], with routine and mood cues linked to automaticity and time or people’s cues predicting behaviour [32]. Our findings extend this by showing how external and internal cues were mobilized to sustain continuity of physical activity and exercise among these older adults.

Technology was identified as a valuable component of habit scaffolding in our study. Participants used apps and wearables for tracking, reminders, and gamified incentives, which reduced cognitive effort and supported routines through goals, monitoring, and prompts. Previous research show that digital behaviour change interventions can significantly increase moderate-to-vigorous physical activity and reduce sedentary time among older adults [33]. Similarly, mobile health (mHealth) apps have been shown to offer promising short-term benefits in terms of increased activity and reduced sitting time, although their long-term impact remains less clear [34]. Complementing this, studies on fall prevention highlight how older adults may develop exercise habits through a dynamic process of identity preservation, planning, and repeated performance, supported by both contextual cues and digital applications [35]. Peng et al. [36] further highlight how digital self-monitoring and behavioural feedback can aid in forming and sustaining health behaviours, particularly when integrated into daily structures. In line with this, the participants in our study did not present technology as a stand-alone driver of habit formation, but as a tool that supported rather than replaced their personally meaningful and adaptable routines.

In addition to these external supports, participants emphasized more internal processes, captured in the sub-theme ‘Cultivating a movement mindset’. Activity was seen as integral to everyday life and wellbeing, driven by health, independence, and positive emotional reinforcement. These experiences resonate with self-determination theory, which suggests that long-term motivation is sustained when behaviours are experienced as self-chosen, competence-enhancing, and connected to one’s sense of self and others [37]. In our study, participants often described movement as something they did both for themselves and for those around them to stay capable, contribute to family life, and maintain social participation. This illustrates how motivation can be both personally meaningful and relationally grounded, combining self-care with a sense of belonging. Participants also reflected on how they made sense of health messages and societal expectations, sorting through advice and scientific claims to find what felt personally relevant and sustainable. This interpretive process aligns with Rhodes and Rebar’s [14] view of physical activity habits as a dynamic system in which reflective and automatic processes coexist and interact, with cognition and affect jointly shaping how habits are reinforced and maintained over time.

Our findings show that sustaining physical activity in later life relied on both strategy scaffolding and internal orientations. Routines, cues, and technologies created stability, while identity, emotions, and meaning provided resilience. This interplay reflects dual-process models where conscious and nonconscious processes work together to sustain physical activity [38]. However, as most evidence stems from younger populations [39], more research is needed on how these processes unfold in later life, where aging brings both challenges and opportunities for continuity in physical activity and exercise.

### Methodological considerations

The study included data from focus groups, a workshop, and a dyadic interview. While this mix of formats partly reflected practical constraints, it can also be seen as a limitation, as group dynamics and levels of interaction may have differed between settings. At the same time, combining interview formats has been shown to enhance data richness and allow different dimensions of experience to emerge. Using a dyadic format may support openness through a shared conversational setting that differs from both individual and group contexts [40].

Even though the inclusion criteria were broad, and the recruitment aimed to capture individuals with varying experiences of physical activity and digital technology use, the sample primarily consisted of individuals with prior experience with regular physical activity and they were technologically adept. This may limit transferability to more sedentary or less technologically confident older adults. At the same time, the results in our study show that even among this relatively active group, maintaining habits for physical activity can be challenging, and habits are not static but change across the life course. That the participants were technologically adept reflects an ongoing generational shift in Sweden, where community-dwelling older adults increasingly use smartphones, tablets, and digital health resources, also among the oldest old [41, 42]. Similar to previous research [43], it is likely that participants with higher eHealth literacy and confidence in managing technology were more inclined to engage in a study of this nature. Still, their diverse attitudes towards technology provided valuable insight into how digital tools can support or hinder habit formation in this population.

In line with Tulle and Palmer’s [44] reflections on qualitative research with active older adults, it is also important to acknowledge that participants may engage in research not only to share their experiences but also to actively position themselves as capable and engaged. Such participation can be seen as a way to counteract stereotypes of passivity in later life, which both enriches and complicates interpretation. The uneven gender distribution across groups could limit transferability to men in general. Homogeneity within groups supported openness and psychological safety [19].

## Conclusion

These findings provide insights into how community-dwelling older adults navigate the challenges of sustaining physical activity, underscoring the interplay between external scaffolding such as cues, routines, and supportive structures, and personal mindsets rooted in identity, emotion, and meaning. Digital solutions emerged as potentially valuable support, not as stand-alone drivers but as tools that can strengthen meaningful and adaptable routines. Further research is needed to determine how sustainable habits for physical activity and exercise can best be supported in later life.

## Supplementary Information

Supplementary Material 1

Supplementary Material 2

## Data Availability

Due to the sensitive and personal nature of the qualitative interview data, transcripts cannot be shared. Anonymized excerpts are included in the article, and further information is available from the corresponding author upon reasonable request.

## Abbreviations

FG: Focus groups
DI: Dyadic interview
W: Workshop
