# Supplementary material for: Staying active through life’s shifting seasons: a qualitative study of community-dwelling older adults’ experiences of habit formation and physical activity in later life
Source: Eur Rev Aging Phys Act. 2025 Nov 27;22:25. doi: 10.1186/s11556-025-00393-8 (PMC12670851; doi:10.1186/s11556-025-00393-8)
Supplement: Supplementary file 1 — Supplementary Material 1 [file 11556_2025_393_MOESM1_ESM.docx]

Appendix 1. The semi-structured interview guide

# **Interview Guide**

# Main questions – Optional follow-up question if further prompting is needed

**1. Welcome and Introduction**
Brief presentation of the moderators. Introduction to the study and a short definition and explanation of key concepts such as physical activity, exercise, behaviour change, and habit formation.

**2. Participant Backgrounds**
To get to know each other:

- please tell us your name and share one personal interest or hobby.

**3. Previous Experiences with Physical Activity**

- Can you tell us about your previous experiences trying to establish a regular routine for exercise or physical activity?
  – Are there any success stories you would like to share?
  – What challenges have you encountered, and how did you deal with them?
- Do you find any difference between developing a habit for general physical activity and for exercise?

**4. Factors Supporting Exercise Habits**

- What do you think is important for building and maintaining a sustainable habit for physical activity?
  – Have you had any experiences that boosted your motivation to stay active?
  – Are there any factors that you feel contribute most to maintaining a routine?

**5. Cues and Triggers for Activity**

- Can you describe any situations or events that usually help or could help you get started with your activity?
  – Are there specific times of day when you feel more likely to be active?
  – Are there certain days of the week that work better for you?
  – Have you noticed places in your home or surroundings where you’re more likely to feel ready to exercise?
  – Are there emotional states or moods that tend to prompt you to move?
  – Do any parts of your daily routine naturally remind you or motivate you to start exercising?

**6. Digital Support for Exercise Habits**

- What are your thoughts about digital tools in supporting your efforts to build and maintain physical activity/exercise habits?
  – Are there any specific features or types of apps you think could be useful?
  – Could you see yourself using digital tools to track your goals and progress? If so, what kind of information would you find valuable?
  – Would digital reminders or triggers be helpful or disruptive?
  ▪ If helpful, when and how should they ideally be delivered?

**7. Other**

- Is there anything we haven’t asked about that you think is important to share on this topic?
