# Supplementary material for: Staying active through life’s shifting seasons: a qualitative study of community-dwelling older adults’ experiences of habit formation and physical activity in later life
Source: Eur Rev Aging Phys Act. 2025 Nov 27;22:25. doi: 10.1186/s11556-025-00393-8 (PMC12670851; doi:10.1186/s11556-025-00393-8)
Supplement: Supplementary file 2 — Supplementary Material 2 [file 11556_2025_393_MOESM2_ESM.docx]

Appendix 2. Overview of the results of the analysis

| **Staying active through life’s shifting seasons** | | | |
| --- | --- | --- | --- |
| Knowing and not knowing the body | Adapting to setbacks and changing conditions | | Reframing physical activity through life transitions |
| **Building the habit scaffold** | | **Cultivating a movement mindset** | |
| Having the right conditions | | Investing in one’s health | |
| Trial and Error: Finding what works | | Mindset of opportunity | |
| A predictable structure | | Movement as a basic need | |
| Social contracts | | The role of commitment, responsibility, and self-regulation | |
| Technology as a feedback system | | Emotional responses | |

The analysis resulted in one overarching theme “Staying active through life’s shifting seasons” which encompassed two interwoven sub-themes: “Building the habit scaffold” and “Cultivating a movement mindset”. Each theme comprised a set of underlying categories.
